# The Hrd1-Mediated ERAD Pathway in Plants: Conserved Principles and Plant-Specific Innovations

**DOI:** 10.3390/ijms27041801

**Published:** 2026-02-13

**Authors:** Jiarui Wu, Peiqi Huang, Jianming Li

**Affiliations:** 1 State Key Laboratory for Conservation and Utilization of Subtropical Agro-Bioresources, South China Agricultural University, Guangzhou 510642, China; jiarui-wu@hkbu.edu.hk (J.W.); hpq@stu.scau.edu.cn (P.H.); 2 Guangdong Key Laboratory for Innovative Development and Utilization of Forest Plant Germplasm, College of Forestry and Landscape Architecture, South China Agricultural University, Guangzhou 510642, China; 3 The Jockey Club STEM Laboratory of Plant Biology, Department of Biology, Hong Kong Baptist University, Kowloon, Hong Kong 999077, China

**Keywords:** endoplasmic reticulum-associated degradation, Hrd1 ubiquitin ligase complex, protein homeostasis, retrotranslocation, E2-E3 pairing

## Abstract

Endoplasmic reticulum-mediated protein quality control (ERQC) safeguards secretory pathway proteostasis by recognizing, retaining, repairing, and removing misfolded proteins, and is therefore essential for plant growth, development, and stress tolerance. This system relies on ER-associated degradation (ERAD), in which irreparably misfolded proteins are first recognized in the ER, then exported across the ER membrane to the cytosol, where they are ubiquitinated by ER membrane-anchored ubiquitin ligases, and subsequently degraded by the cytosolic proteasome. Studies in yeast and mammals have defined several conserved ERAD branches, including a multiprotein ERAD complex centered on the polytopic ER membrane E3 ligase HMG-CoA reductase degradation protein 1 (Hrd1), which integrates substrate recognition, membrane retrotranslocation, ubiquitin conjugation, and cytosolic extraction. Recent advances in Arabidopsis show that plants retain the core Hrd1 ERAD architecture while incorporating additional regulatory elements that adapt this machinery to plant-specific physiological demands. Genetic and biochemical analyses of misfolded receptor kinases and engineered substrates have uncovered conserved and plant-specific components of the plant Hrd1 complex, revealing how the plant ERAD pathway integrates ERQC with hormone signaling, stress adaptation, immune responses, and growth regulation. This review synthesizes recent advances in plant ERAD research and highlights key conceptual and mechanistic questions that remain to be resolved.

## 1. Introduction

Plants harness solar energy to assimilate atmospheric carbon dioxide, producing oxygen and organic matter that sustain global ecosystems [1,2]. Their growth and productivity are profoundly influenced by abiotic factors such as temperature, drought, salinity, and nutrient availability, which define both plant distribution and agricultural yield [3,4]. However, because plants are sessile organisms that cannot escape these adverse conditions, they are inherently vulnerable to environmental fluctuations. Exposure to abiotic stress frequently disrupts the error-prone protein folding process by perturbing the ER environment required for efficient folding, including ER redox balance, calcium fluctuation, and energy supply, thus leading to the accumulation of misfolded proteins and triggering a cellular condition known as ER stress [5,6]. If unmitigated, ER stress disrupts protein homeostasis, impairs plant growth and development, and can ultimately result in cell death [6,7]. To preserve proteome integrity and cellular homeostasis, eukaryotic cells have evolved several interconnected regulatory modules of the ER proteostasis network, including ER protein quality control (ERQC) that recognizes, retains, and repairs misfolded and improperly assembled proteins, and ER-associated degradation (ERAD), unfolded protein response (UPR), and autophagy [8,9]. Among these, ERAD provides the most direct strategy of alleviating ER stress by recognizing irreparable misfolded proteins and targets them for ubiquitin- and proteasome-mediated degradation [9,10].

**Figure 1:**
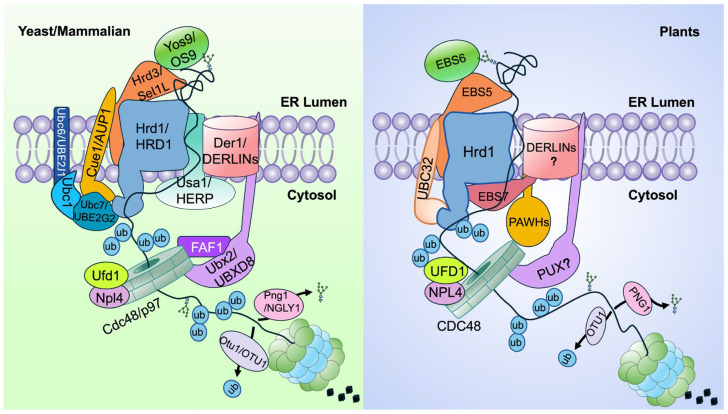
A model of the Hrd1-mediated ERAD pathway. This model compares the Hrd1 ERAD complex in yeast/mammalian cells and plants. Core elements or steps, such as substrate-recruitment factors Yos9/OS9/EBS6 and Hrd3/Sel1L/EBS6, the ER membrane-embedded E3 ligase Hrd1 with its E2 conjugase(s), DERLIN-supported retrotranslocation, Cdc48-driven substrate extraction, post-extraction Otu1/PNG1-mediated substrate processing, and cytosolic proteasome-mediated proteolysis, are broadly conserved. In yeast and mammals, Hrd3/Sel1L stabilizes Hrd1, whereas in plants this role is fulfilled by the plant-specific factors EBS7 and PAWH1/PAWH2. Hrd1 also employs distinct adaptors in different lineages to engage their respective E2 enzymes. Although DERLIN-family proteins are conserved, their assembly state and mode of coupling to Hrd1 likely differ among eukaryotes. Retrotranslocation relies on the Cdc48-Ufd1-Npl4 complex, whose recruitment onto the Hrd1 ERAD machinery varies among different species.

Genetic and biochemical studies in yeast and mammals have elucidated the core architecture and mechanistic logic of ERAD [11]. A typical ERAD machinery is organized around an ER-membrane-anchored E3 ubiquitin ligase whose catalytic domain faces the cytosol, together with several accessary factors, operating through four interdependent steps: (1) substrate recognition and recruitment in the ER, (2) retrotranslocation across the ER membrane, (3) ubiquitination on the cytosolic face of the ER membrane, and (4) proteasome degradation in the cytosol [12]. In yeast, two major ERAD branches have been defined. The HMG-CoA reductase degradation 1 (Hrd1) complex (Figure 1), centered on a six-pass ER membrane-anchored E3 ligase, mediates the degradation of luminal and membrane ERAD substrates (ERAD-L and ERAD-M) carrying luminal or membrane-localized folding defects, respectively. In contrast, the Degradation of alpha-2 10 (Doa10) complex, built around a 14-pass ER membrane-embedded E3 ligase, targets ERAD-C substrates carrying cytosolic misfolding lesions [11,13]. In mammals, the ERAD system is diversified into multiple branches, each centered on a distinct ER membrane E3 ligase that targets specific classes of misfolded or unwanted proteins. Among them, the HRD1 module (Figure 1) and the transmembrane ER-bound RING finger protein 4 (TEB4)/Membrane-Associated Ring-CH 6 (MARCH6) complex are functional analogs of the yeast Hrd1 and Doa10 branches, respectively, underscoring both the evolutionary conservation of ERAD principles and the increased complexity of ERAD in multicellular organisms [11,14].

The yeast/mammalian Hrd1 complex comprises multiple accessory factors that coordinate the four steps of the ERAD pathway (Figure 1) [12]. In the ER lumen, the lectin yeast homolog of Osteosarcoma Amplified 9 (Yos9)/OS9 recognizes the conserved ERAD-specific asparagine-linked glycan (N-glycan) signal on terminally misfolded glycoproteins [15,16,17,18], while Hrd3 and its mammalian homolog Suppressor/Enhancer of Lin-12-like (Sel1L) bind exposed hydrophobic residues on misfolded proteins [13,17,19]. This dual-recognition system ensures that irreparably misfolded substrates are selectively delivered to the Hrd1 complex [20]. Within the ER membrane, the E3 ubiquitin ligase Hrd1/HRD1 cooperates with the four-pass membrane protein known as Degradation in the endoplasmic reticulum 1 (Der1)/Der1-like protein (DERLIN) [21,22], assisted by the scaffolding protein U1-snp1 Associating 1 (Usa1)/Homocysteine-induced endoplasmic reticulum protein (HERP) [23,24,25,26], to mediate the retrotranslocation of ERAD substrates across the lipid bilayer. Although mammalian DERLINs are known to be involved in retrotranslocating ERAD substrates [27,28], whether or not this process requires HRD1 remains unanswered. On the cytosolic face, Hrd1 functions primarily with the E2 enzyme Ubiquitin-Conjugating enzyme 7 (Ubc7), assisted by Ubc1, to catalyze substrate ubiquitination [29]. Both E2s are soluble cytosolic enzymes recruited to the ER membrane by Coupling of ubiquitin conjugation to ER degradation 1 (Cue1) [30], an ER membrane-anchored adaptor that binds and activates Ubc7 [31]. In contrast, the mammalian HRD1 works with Ubiquitin-conjugating enzyme E2 J1 (UBE2J1), one of the two mammalian homologs of the yeast Ubc6, and UBE2G2, one of the two mammalian Ubc7 homologs, to ubiquitinate HRD1 clients [32,33]. The energy-dependent extraction of luminal and membrane substrates is driven by the Cell division cycle 48 (Cdc48) complex, composed of the AAA-type ATPase Cdc48/valosin-containing protein [VCP, also known as p97 for a protein product of *M*_r_~90,000] and its two cofactors Ubiquitin fusion degradation 1 (Ufd1) and Nuclear protein localization protein 4 homolog (Npl4). Such an ATP-dependent substrate-extraction complex is recruited to the Hrd1 complex through UBX domain-containing protein 2 (Ubx2)/UBX Domain-containing protein 8 (UBXD8) [34,35,36]. In the cytosol, the Ufd1–Npl4–Cdc48 complex escorts ubiquitinated substrates to the proteasome, where Ovarian tumor domain-containing deubiquitinating enzyme (Otu1)/OTUD1 mediates deubiquitination, and Peptide-N-glycanase 1 (Png1)/N-glycanase 1 (NGLY1) removes N-glycans, thereby facilitating efficient substrate entry into the proteasome’s catalytic chamber for complete proteolysis [12,37].

Compared with the extensive studies in yeast and mammals, research on the plant ERAD pathway has lagged behind, largely due to the historical lack of convenient genetic and biochemical systems [8]. However, recent advances, particularly genetic analyses in *Arabidopsis thaliana* combined with biochemical and proteomic approaches, have led to the identification of the major components of the Arabidopsis Hrd1 ERAD system (Figure 1 and Table 1) [8,38]. These include not only evolutionarily conserved core elements shared with yeast and mammals but also plant-specific factors that appear unique to the green lineages. Biochemical studies have further revealed several distinctive features of the plant ERAD machinery, underscoring its evolutionary innovation and physiological relevance. This review summarizes the discovery, biochemical characterization, and physiological functions of known components of the plant Hrd1-ERAD pathway, highlighting both conserved and lineage-specific mechanisms. We hope that this review will stimulate further research to fill the remaining knowledge gaps and exploit the powerful plant genetic systems available today to address outstanding questions in the ERAD field.

## 2. The Hrd1-Mediated ERAD Pathway in Plants

The ERAD pathway is evolutionarily conserved in plants [8,38]. Early evidence from tobacco protoplasts and transgenic tobacco plants revealed that some heterologously expressed fusion proteins were retained and degraded in the ER [39,40,41,42,43]. A key model substrate was the catalytic A subunit (RCA) of ricin, a highly toxic ribosome-inactivating plant toxin from the seeds of caster bean *Ricinus communis* L. [40,41]. When expressed in tobacco protoplasts, RCA was retrotranslocated to the cytosol and degraded via a Cdc48-dependent proteasomal pathway [40,44,45]. Similarly, heterologously expressed mutant variants of the protein product of the barley Mildew resistance locus o (MLO), which encodes a plasma membrane (PM)-localized seven transmembrane domain-containing protein [46], were ubiquitinated and removed via a similar ERAD-like route [47]. These artificial ERAD substrates demonstrated that plant cells possess a functional ERAD mechanism for eliminating misfolded glycoproteins.

Genetic studies of the Arabidopsis *brassinosteroid-insensitive 1-5* (*bri1-5*) and *bri1-9* dwarf mutants provided the first endogenous ERAD substrates in a genetic model system: two mutant variants (bri1-5 and bri1-9) of the brassinosteroid (BR) receptor BRI1 are retained in the ER by an N-glycan-mediated ERQC system and degraded through an ERAD pathway [10]. A forward-genetic suppressor screen of *bri1-9* carrying a Ser^662^-Phe substitution in the BR-binding domain, which was originally designed to identify components of the BR signaling pathway, identified loss-of-function mutations in *EMS-mutagenized bri1 suppressor 1* [*EBS1* encoding the Arabidopsis homolog of the mammalian UDP-glucose:glycoprotein glucosyltransferase (UGGT)] and *EBS2* [encoding calreticulin 3 (CRT3), one of the three Arabidopsis homologs of the mammalian CRT/calnexin (CNX) proteins] [48,49]. These studies revealed that the Ser^662^-Phe mutation of bri1-9 does not abolish BR binding but instead introduces a subtle folding defect that is recognized by the N-glycan-dependent ERQC cycle, causing its ER retention and its BR-insensitive dwarfism phenotype. Disruption of ERQC in *ebs1 bri1-9* or *ebs2 bri1-9* mutants allowed structurally imperfect yet signaling-competent bri1-9 to reach the PM, thus suppressing the growth defects of the *bri1-9* mutant [48,49]. Subsequent biochemical analyses identified bri1-5, carrying a Cys^69^-Tyr substitution in the N-cap of its extracellular domain, as a second ER-retained mutant variant of the BR receptor [50]. Mutations that impair their ERAD pathway result in the accumulation of bri1-5 and bri1-9 in the ER to levels that saturate their corresponding retention mechanisms, thereby permitting a small fraction of ER-retained mutant bri1 proteins to “leak” to the PM, where bri1-5 and bri1-9 bind BRs, thus activating BR signaling to restore normal growth of the two BR receptor mutants [10].

Suppressor screens and reverse-genetic analyses of the two endogenous substrates have demonstrated that plants conserve the canonical N-glycan-based ERAD signal and its generation mechanism. This ERAD signal is a specific N-glycan carrying an exposed α1,6-mannose (α1,6-Man) residue that marks misfolded glycoproteins for ERAD [12,16]. EBS3 and EBS4 encode ER-localized mannosyltransferases required for the proper assembly of the N-glycan precursor [51,52,53,54,55], and their loss-of-function mutations produce truncated N-glycans lacking the canonical α1,6-Man residue, thus effectively blocking the ERAD of the two mutant BR receptors. By contrast, reversal of bri1 (IRB1)/mannosidase 5 (MNS5) and MNS4 are the functionally redundant ER-localized α1,2-mannosidases that cleave the terminal α1,2-Man residue to expose its protected α1,6-Man residue, and their mutations abolish the enzyme activity required to generate the N-glycan ERAD signal [56,57,58]. Together, these results established that the biochemical logic of N-glycan-based misfolded-protein marking is conserved in plants.

Genetic and proteomic investigations also defined the core components of the Arabidopsis Hrd1-mediated ERAD machinery (Figure 1). They include EBS5 (also known as AtHrd3/AtSel1L for its homology to the yeast Hrd3 and mammalian Sel1L) [59,60] and EBS6 (also known as AtOS9 for its sequence similarity to yeast Yos9 and mammalian OS9) [61,62], which mediate substrate recognition and recruitment, while reverse genetics confirmed that Hrd1A/Hrd1B function redundantly as the core E3 ubiquitin ligases [60] (Table 1). A forward-genetic hit, EBS7, encodes a plant-specific ER membrane protein required for Hrd1 stability [63], while a proteomic experiment further identified two Hrd1-binding ER membrane proteins, protein associated with hrd1 1 (PAWH1) and PAWH2, each containing a conserved altered inheritance of mitochondria protein 24 (AIM24) domain forming a compact helical scaffold [64]. Although AIM24 proteins are widely distributed across plants, animals, bacteria and archaea [65], PAWH1/2 represent a plant-adapted module that stabilizes the Hrd1 complex.

Other experimental approaches implicated additional components in the plant Hrd1-mediated ERAD pathway. In rice, overexpression or RNAi-mediated silencing of an *Oryza sativa Der1* homolog (*OsDER1*) activates the rice UPR pathway and causes hypersensitivity to ER stress, suggesting a conserved role in a plant ERAD pathway, although its specific function in ERAD remains unsolved [66]. Phenotypic analysis of Arabidopsis mutants coupled with a transient tobacco expression system using dominant-negative mutant transgenes supports the involvement of the Cdc48 AAA+-type ATPase in the substrate extraction step and contribution of the deubiquitinase Otu1/2 and PNG1 in the post-extraction substrate processing, although definitive genetic evidence, particularly for Otu1/2 and PNG1, is still lacking [67,68]. An earlier study also implicated several Arabidopsis heavy metal-associated isoprenylated plant proteins (HIPPS) in a post-retrotranslocation event, although these functions await validation by robust biochemical assays. Collectively, these findings suggest that the post-retrotranslocation phases of Hrd1-mediated ERAD, including substrate dislocation, ubiquitin chain editing, N-glycan processing, and proteasomal degradation, are broadly conserved in plants, while key mechanistic details remain to be established.

Altogether, these discoveries establish the two Arabidopsis dwarf mutants as a powerful genetic model for the mechanistic dissection of the ERQC and ERAD pathway, revealing a conserved EBS5/EBS6-Hrd1 core machinery elaborated by plant-specific factors such as EBS7, PAWH1/2, and possibly HIPPS, which together integrate ER proteostasis with growth, hormone signaling and stress responses.

## 3. Regulation of the Hrd1-ERAD Complex

The capacity of an ERAD mechanism must be carefully balanced with folding demand in the ER to avoid the premature degradation of newly synthesized or incompletely folded proteins that are still engaging chaperone-assisted folding or refolding attempts. Across eukaryotes, this balance is achieved in part through “ER tuning”, a conserved mechanism in which selected ERAD components are themselves degraded under non-stress conditions to prevent excessive proteolysis but stabilized during ER stress to transiently increase ERAD activity. In mammals, OS9, ER degradation-enhancing alpha-mannosidase-like protein 1 (EDEM1, a homolog of Arabidopsis MNS4/MNS5), and the E2 enzyme UBE2J1 are well-established ER-tuning substrates whose turnover is Hrd1-dependent [69,70,71,72,73]. In Arabidopsis, this conserved principle is exemplified by UBC32, which is continuously degraded under basal conditions but stabilized during ER or abiotic stress to enhance ERAD capacity and stress resilience [70]. Although an earlier study using transgenically expressed EBS6/AtOS9 suggested that EBS6/AtOS9 is similarly regulated [74], a recent study indicated that the endogenous EBS6/AtOS9 is relatively stable [75]. This discrepancy may arise from stoichiometric imbalance caused by non-physiological overexpression, resembling the reduced stability of the endogenous EBS6/AtOS9 in the *ebs5* mutant [62]. Accordingly, these observations suggest that caution is warranted when inferring endogenous degradation-based regulatory mechanisms from transgenic protein behavior. Further studies will be required to determine whether other plant ERAD factors, including MNS4 and MNS5/IRB1, are also subject to ER-tuning regulation.

In plants, the mechanism governing Hrd1 stability itself has diverged sharply from those described in yeast and mammals, in which the luminal scaffold Hrd3/Sel1L stabilizes Hrd1. Mutations of the yeast *Hrd3* lead to Hrd1 degradation in a Usa1p-dependent manner, while knocking out *Sel1L* in mice markedly reduces the abundance of HRD1 [25,71,76]. By contrast, in Arabidopsis, loss of the Hrd3/Sel1L homolog EBS5 has little effect on Hrd1 abundance but reduces the protein abundance of EBS6/AtOS9 [62,64]. Instead, Hrd1 stability is maintained by two plant-specific components: EBS7 and PAWH1/PAWH2 [63,64]. Notably, these components are mutually dependent; loss of EBS7 destabilizes PAWH1/2 and Hrd1, and loss of PAWH1/2 destabilizes EBS7 and Hrd1, while loss of Hrd1A/B has little effect on the stability of EBS7 and PAWHs [64]. This creates a plant-specific auto-regulatory circuit that maintains the stoichiometric integrity of the Hrd1 complex independently of Hrd3/Sel1L.

## 4. The Hrd1-Associated E2 Enzymes

In yeast and mammals, Hrd1/HRD1 operates with paired E2 enzymes to ubiquitinate committed ERAD substrates [77]. The yeast Hrd1 primarily employs Ubc7, with Ubc1 contributing to ubiquitination of selected substrates, whereas the ER-anchored Ubc6 functions mainly with the Doa10 branch [78,79,80,81]. Because both Ubc7 and Ubc1 are cytosolic, they are recruited to the Hrd1 complex by Cue1, which serves as both a membrane tether and an allosteric enhancer [30,82]. The mammalian Hrd1 system utilizes a Ubc6 homolog, UBE2J1, in addition to a Ubc7 homolog, UBE2G2, with diverged recruitment modules [32,83]. The Ubc6-type UBE2J1 is recruited by Sel1L [84] while the Ubc7-type UBE2G2 is recruited by ancient ubiquitous protein 1 (AUP1) [85,86], which additionally links ERAD capacity to lipid-droplet homeostasis [87].

In plants, the identity and recruitment of the E2 enzymes that function with the Hrd1 E3 ligase remain incompletely defined. Arabidopsis encodes multiple E2s related to Ubc6- (UBC32/33/34), Ubc1 (UBC27), Ubc7 (UBC7/13/14) [88,89,90], yet no Cue1- or AUP1-like recruitment factor has been identified. Among these, the ER-anchored UBC32 contributes to Hrd1-dependent degradation of misfolded bri1 proteins, but its loss only partially impairs ERAD and incompletely suppresses the *bri1* dwarfism, implying functional redundancy with additional E2s [91]. Transient-expression assays suggest that UBC33 and UBC34 may also support Hrd1-mediated degradation of artificial substrate, although their genetic contribution to endogenous ERAD substrates requires validation [92]. Likewise, the possible involvement of Ubc7-type (UBC7/13/14) or Ubc1-type (UBC27) enzymes in Hrd1-mediated ERAD remains largely untested despite a recent report of hypersensitivity to ER stress of an Arabidopsis mutant lacking all three Ubc7-type E2 enzymes [93]. The absence of a Cue1/AUP1-like E2-recruitment module suggests that plants may have evolved a distinct E2-E3 assembly mechanism, representing a plant-specific reconfiguration of the otherwise conserved Hrd1-ERAD system. Notably, these E2 enzymes are not restricted to the canonical Hrd1-ERAD pathway but instead participate in multiple ubiquitin-dependent degradation pathways that regulate growth, development, and stress tolerance [89,93,94,95,96,97,98,99,100,101,102,103].

## 5. The Retrotranslocon of the Hrd1 Pathway

Retrotranslocation of misfolded ER proteins into the cytosol is a defining feature of ERAD [104] and was among the earliest ERAD events to be explored in plant systems [40,44,105]. However, despite this early attention, the molecular identity of the plant ERAD retrotranslocon remains poorly understood. In yeast, the Hrd1 retrotranslocon operates through an Hrd1-Der1 membrane module in which Hrd1 and Der1 each contribute a hydrophilic half-channel positioned side-by-side within a locally thinned membrane, forming mutually facing lateral gates that allow a loop of an ERAD-L substrate to insert and traverse the ER membrane [106]. In mammals, DERLIN1 associates with p97/VCP to form multiple channel assemblies that likely adapt to substrate sizes and structural states [107,108,109]. Thus, although DERLIN-family proteins are conserved across eukaryotes, their assembly state and mode of coupling to Hrd1 diverge among eukaryotes.

Plants possess multiple *DERLIN* genes, which were first identified in maize and Arabidopsis nearly two decades ago [110], but their specific roles in Hrd1-dependent ERAD remain unresolved. Early genetic and expression studies suggested that plant DERLINs participate in ERQC [110,111], but direct mechanistic evidence linking any plant DERLIN to the Hrd1 ERAD complex remains rather limited. A recent report proposed that rice OsDER1 contributes to ERAD [66], but the phenotypic and biochemical data are not yet definitive, and whether OsDER1 directly engages Hrd1 or functions in a parallel membrane-remodeling process requires further investigation. Given structural and evolutionary parallels to yeast and mammals, it is reasonable to hypothesize that one or more of the plant DERLINs collaborates with the plant Hrd1 E3 ligase to form the core retrotranslocon [10], but whether plants employ a yeast-like Hrd1-Der1 hybrid conduit, a mammalian-like DERLIN oligomeric channel, or a plant-specific mechanism, remains an open and central question in plant ERAD biology (Figure 1).

## 6. Recruitment of the Cdc48 Complex

As discussed above, the overall sequence of the plant Hrd1 ERAD pathway, substrate recognition and recruitment, Hrd1-mediated ubiquitination, retrotranslocation, Cdc48-driven extraction, and proteasomal degradation are highly conserved compared to those of yeast and mammals [8,9]. However, while the requirement of Cdc48/p97 for retrotranslocation and post-retrotranslocation processing is established in plants [44,47,112], how the Cdc48 complex is recruited to the Hrd1 machinery remains unresolved. In yeast, this recruitment is mediated by Ubx2, a tail-anchored ER protein whose UBX (ubiquitin-regulatory X) domain binds Cdc48 and whose UBA (ubiquitin-associated) domain associates with ubiquitinated substrates and Hrd1 [34,35]. In mammals, this recruitment function is shared between UBXD8 (also known as FAF2 for Fas-associated factor family member 2), an ER-anchored adaptor linking p97/VCP to the Hrd1-Sel1L complex, and FAF1, a soluble UBX protein that regulates substrate selection and p97/Cdc48 activity (Figure 1) [36,113,114]. Thus, in yeast and mammalian cells, a membrane-associated UBX adaptor is central to bridging ubiquitination and extraction.

In plants, no direct Ubx2/UBXD8 equivalent has yet been identified. Arabidopsis encodes a plant UBX domain-containing protein (PUX) family of 15 members [10,115], many of which interact with Cdc48A, but only PUX10 contains a C-terminal transmembrane anchor, making it the sole membrane-tethered UBX protein and therefore the most structurally plausible candidate for recruiting Cdc48A to the Hrd1-EBS5/EBS6 complex [112]. Consistent with its membrane-tethered nature, PUX10-GFP expressed in stable transgenic Arabidopsis lines or transiently in tobacco leaves localizes to lipid droplets, chloroplasts, and the ER [116]. In line with this localization pattern, PUX10 was initially characterized at lipid droplets, where it recruits Cdc48A to mediate oleosin turnover and lipid-droplet remodeling [116,117], and was recently shown to act in a chloroplast-associated degradation (CHLORAD) pathway by recruiting Cdc48A to the chloroplast outer envelope for degrading the TOC (translocon at the outer chloroplast membrane) complex [118]. However, PUX10 has not been identified in forward-suppressor screens of *bri1-5* or *bri1-9*, and no biochemical interaction with the Hrd1 assembly has been demonstrated, leaving its role in ERAD unproven. Other PUXs, including PUX3, PUX4, and PUX5, function in nuclear-associated protein degradation [119], highlighting functional diversification within the family. Thus, although the mechanistic logic of coupling Hrd1 ubiquitination to Cdc48-powered protein extraction is conserved [67], the identity of the Cdc48-recruiter in the plant Hrd1-mediated ERAD pathway remains unknown, and whether the ER membrane-anchored PUX10 or a soluble PUX serves as the Cdc48 recruiter to the Hrd1 ERAD machinery is a key open question that needs to be investigated (Figure 1).

## 7. The Plant Hrd1 Does Not Degrade HMGCR

In yeast and mammals, 3-hydroxy-3-methylglutaryl coenzyme A (HMG-CoA) reductase (HMGCR), a key rate-limiting enzyme of the sterol biosynthetic pathway, is a classical substrate of the Hrd1 ERAD pathway, directly linking ERAD to sterol homeostasis [120,121]. Notably, the major components of the yeast Hrd1 complex were originally identified and named for their roles in HMGCR degradation (Hrd1-3 for HMG-CoA reductase degradation proteins 1-3) [122]. The Hrd1–Hrd3/Sel1L machinery promotes sterol-dependent ubiquitination and retrotranslocation of HMGCR, illustrating how lipid metabolism is embedded within the core Hrd1 ERAD system in yeast and mammals [123,124,125]. In plants, however, HMGCR degradation occurs outside the Hrd1 pathway. In *Medicago*, jasmonate-induced HMGCR turnover is mediated by Makibishi (MKB1), a member of the conserved RING-membrane-anchored (RMA)-type E3 ubiquitin ligase family [126], while in Arabidopsis and maize, membralin-like proteins, high sterol ester 1 (HISE1 in Arabidopsis) and narrow leaf and dwarfism 1 (NLD1 in maize), interact with ring finger protein 185 (RNF185, another RMA-type E3 ligase) to promote HMGCR degradation [127,128]. Although the homologous membralin-RNF185 complex exists in mammals to degrade membrane proteins [129], its role in HMGCR turnover has not been demonstrated, suggesting that plants uniquely repurposed this ERAD branch for sterol metabolic regulation. Thus, HMGCR degradation is ERAD-regulated across eukaryotes, but plants have shifted this function away from the conserved Hrd1 ERAD branch to a membralin-based pathway, reflecting an evolutionary rewiring that integrates lipid metabolism with plant-specific physiological contexts.

## 8. Physiological Functions of the Plant Hrd1-ERAD System

Although originally identified through yeast genetic studies of the regulated turnover of HMGCR, the Hrd1-mediated ERAD pathway was later established as a core ERQC mechanism for eliminating irreparably misfolded proteins and is increasingly recognized in recent years as a central regulatory system that coordinates growth, hormone balance, stress adaptation, and immunity in plants [9,130]. Because many cell surface-localized receptors, hormone and metabolite transporters, ion channels, and apoplastic enzymes must fold and assemble in the ER, the Hrd1 ERAD system does not simply protect proteostasis; it determines which signaling and metabolic regulators reach functional destinations, thereby tuning developmental and environmental responses.

In growth and development, Hrd1-ERAD functions as a hormone and signaling homeostasis gate. For BR signaling, this system adjusts BRI1 receptor dosage and ER export competence [38]. A parallel mechanism operates in auxin biology, where ERAD controls the levels of PILS (PIN-LIKES), ER-localized auxin transport regulators that modulate cytosolic versus ER auxin pools upstream of PIN-FORMED (PIN)-mediated transport [131]. Cytokinin homeostasis is likewise ERAD-gated: the cytokinin-degrading enzyme cytokinin oxidase 1 (CKX1) is selectively targeted for ERAD through the Hrd1-ERAD pathway [132,133]. ERAD also regulates the HAESA (HAE)/HAESA-Like 2 (HSL2) receptor dosage to control floral abscission [54]. Together, these studies demonstrate that the Hrd1 ERAD pathway constitutes a unified regulatory axis that coordinates receptor maturation, intracellular hormone distribution, and hormone turnover to calibrate developmental signaling thresholds.

Hrd1-ERAD is also fundamental to stress resilience. UBC32 undergoes dynamic turnover, rapidly degraded under basal conditions and stabilized during ER stress, to scale ERAD capacity during salt, heat, drought, and oxidative stress [70,91]. Consistently, mutations in core components of the Hrd1 ERAD pathway increase ER stress sensitivity and compromise stress tolerance [59,60,61,62,63,64,92,101,134,135,136]. For example, loss-of-function mutations in EBS5/AhHrd3, EBS6/AtOS9, Hrd1s, EBS7 or PAWHs dramatically compromise seedling survival on high-salt growth medium (59–64). Similarly, simultaneous elimination of UBC32 and its two close homologs inhibits seed germination and seedling growth under salt/osmotic stress conditions (92, 101, 135). Recent genetic studies further revealed that loss of both Hrd1- and Doa10 additively enhances thermotolerance while constitutively activating both UPR and cytosolic protein response, demonstrating that ERAD actively modulates heat-stress signaling rather than acting solely as a disposable mechanism [137]. In addition, mutations in Hrd1 and EBS5/AtHrd3 also cause selenate-hypersensitivity [138], indicating that ERAD prevents misfolded or selenium-binding proteins from triggering chronic ER stress. This buffering function is equally critical during secretory and energy-intensive developmental stages. In Arabidopsis, Hrd1A and Hrd1B are required to maintain cuticular wax and cutin biosynthesis during epidermal differentiation [139]. In rice endosperm, a conserved OsHrd1 pathway removes misfolded native storage proteins generated during high metabolic flux, thereby maintaining protein body organization and preventing chronic UPR activation during grain filling [66,140]. Together, these findings highlight a conserved function of the Hrd1-ERAD pathway in preserving ER homeostasis during both environmentally and metabolically demanding stages of plant growth.

In immunity, ERAD contributes to both pattern-triggered immunity (PTI) and effector-triggered immunity (ETI) [9]. When ER folding capacity is limiting, plant immunity receptors, such as Elongation Factor-Thermal Unstable (EF-Tu) receptor (EFR) and suppressor of bir1-1 (SOBIR1), fail to mature and are redirected toward degradation, diminishing basal defense signaling [8,62,141]. Conversely, resistance-associated MLO mutant variants require Hrd1 and Cdc48 for degradation [47], indicating that membrane immune surveillance relies on a functioning ERAD axis [142]. The tobacco Hrd1 directly ubiquitinates viral triple-gene-block movement proteins, restricting systemic infection and underscoring a direct antiviral role for ERAD machinery [143]. The ER-associated E2 enzymes also contribute to immune competence: UBC32 and its two closest homologs in Arabidopsis and tomato [92], and their rice homolog OsUBC45 [96], promote defense by regulating immune protein turnover. Major resistance genes that enhance blast disease resistance typically lead to yield penalties in rice [144], but OsUBC45 overexpression confers broad-spectrum disease resistance and increased grain yield in rice by promoting the degradation of glycogen synthase kinase 3 (OsGSK3) and aquaporin OsPIP2;1, which negatively regulate grain size and PTI, respectively, thereby supporting sustainable rice production [96]. Together, these findings establish the Hrd1-ERAD complex as central regulators of immune receptor stability and pathogen resistance.

Therefore, the HRD1 ERAD machinery functions as an important proteostatic rheostat that links ER folding capacity with receptor signaling, hormone metabolism, stress adaptation, and immune responsiveness. Although our current knowledge of endogenous ERAD substrates in plants remains limited, existing genetic and functional evidence indicates that this system allows plants to dynamically adjust growth-defense balance and maintain cellular homeostasis during environmental and developmental challenges.

## 9. Conclusions and Future Perspective

The plant Hrd1-mediated ERAD pathway is built upon a conserved mechanistic core but features green life-specific regulatory innovations and adaptations. In yeast, mammals, and plants, ER-retained misfolded proteins are recognized and recruited to the Hrd1 E3 ligase through an OS9-family lectin in cooperation with the Hrd3/Sel1L/EBS5 scaffold. This conserved substrate-recognition module funnels ERAD clients into the retrotranslocation and Cdc48-proteasome degradation route. Likewise, “ER tuning” of the membrane-anchored Ubc6/UBC32-type E2 enzyme and other ERAD components appears broadly shared. However, the stability and self-regulation of Hrd1 differ markedly in plants. Whereas Hrd1 abundance in yeast and mammals is largely scaffold-stabilized by Hrd3/Sel1L, plants employ a green life-specific regulatory module, EBS7, together with PAWH1/PAWH2, to stabilize the membrane-anchored E3 ligase, which could potentially regulate the ERAD capacity according to physiological demand and environmental signals. Although Cdc48 is essential for substrate extraction, the adaptor linking Hrd1 to the Cdc48-Ufd1-Npl4 complex remains unidentified, and the UBX protein PUX10 is a promising but still unconfirmed candidate. Similarly, the oligomeric organization and functional engagement of DERLINs in the retrotranslocation step remain to be defined. Finally, plants have partitioned HMGCR degradation into an Hrd1-independent ERAD pathway, underscoring that ERAD evolution in plants supports metabolic and signaling priorities distinct from those in yeast and mammalian systems.

These mechanistic differences are mirrored by the physiological roles that the Hrd1-ERAD pathway fulfills in plants. Beyond safeguarding secretory proteome fidelity, Hrd1-mediated protein degradation influences receptor quality control, sterol metabolism, protein trafficking competency, and the balance between growth and stress tolerance. Defects in components of the Hrd1 ERAD machinery alter plant growth and development, compromise immunity and abiotic stress resilience, and sensitize the ER to misfolded-protein burden, illustrating how ERAD operates as a critical integrator of cellular homeostasis.

Looking ahead, resolving the unique features of the plant Hrd1-ERAD pathway will require proteomics, cryo-electron microscopy, substrate-trapping variants, proximity labeling, and comparative evolutionary analyses across land plant lineages. Key outstanding questions include: What additional E2 enzymes cooperate with Hrd1, and how is their recruitment to the Hrd1 machinery achieved? What adaptor proteins bridge Hrd1 to the Cdc48-Ufd1-Npl4 complex? How are DERLINs arranged on the ER membrane into functional retrotranslocons? Equally important is understanding how hormonal and environmental signals regulate the ERAD capacity in vivo, particularly in the context of a rather limited catalog of endogenous physiological substrates. Elucidating these mechanisms will deepen our understanding of the ER proteostasis network, clarify how plants balance growth and stress response at the molecular level, and offer new opportunities to engineer crop resilience through targeted modulation of the plant ERAD machinery.

## Figures and Tables

**Table 1:** Components of the Hrd1-ERAD pathway in yeast, mammals, and Arabidopsis.

Yeast Component	Mammalian Component	Arabidopsis Component	Arabidopsis Gene ID
Hrd3	SeI1L	EBS5/AtHrd3/AtSel1L	AT1G18260
Yos9	OS9, XTP3-B	EBS6/AtOs9	AT5G35080
Der1	DERLIN1	DERLIN1	AT4G29330
	DERLIN2	DERLIN2.1	AT4G21810
	DERLIN3	DERLIN2.2	AT4G04860
Hrd1	Hrd1	Hrd1A	AT3G16090
		Hrd1B	AT1G65040
-	-	EBS7	AT4G29960
-	-	PAWH1	AT4G17420
		PAWH2	AT5G47420
Ubc1	UBE2K	UBC27	AT5G50870
Ubc6	UBE2J1	UBC32	AT3G17000
		UBC33	AT5G50430
		UBC34	AT1G17280
Ubc7	UBE2G2	UBC7	AT5G59300
		UBC13	AT3G46460
		UBC14	AT3G55380
Cue1	AUP1	-	-
Ufd1	UFD1	UFD1	AT2G21270
			AT2G29070
			AT4G38930
			AT4G15420
Npl4	NPL4	NPL4	AT2G47970
			AT3G63000
Ubx2	UBXD8	PUX?	?
Otu1	OTUD1	OTU1	AT2G28120
		OTU2	AT1G50670
Png1	NGLY1	PNG1	AT5G49670
Cdc48	VCP/p97	CDC48A	AT3G09840
		CDC48B	AT2G03670
		CDC48C	AT3G01610

?: a promising but still unconfirmed candidate; and -: lacking a homolog.

## Data Availability

No new data were created or analyzed in this study. Data sharing is not applicable to this article.
